# Inhibition of ERα/ERK/P62 cascades induces “autophagic switch” in the estrogen receptor-positive breast cancer cells exposed to gemcitabine

**DOI:** 10.18632/oncotarget.10363

**Published:** 2016-07-01

**Authors:** Peng Shen, Ming Chen, Mengye He, Luoquan Chen, Yinjing Song, Peng Xiao, Xiaopeng Wan, Feng Dai, Ting Pan, Qingqing Wang

**Affiliations:** ^1^ Department of Medical Oncology, First Affiliated Hospital, Zhejiang University School of Medicine, Hangzhou 310003, PR China; ^2^ Institute of Immunology, Zhejiang University School of Medicine, Hangzhou 310058, PR China

**Keywords:** gemcitabine, breast cancer, autophagy, estrogen receptor, P62

## Abstract

Several clinical trials revealed that estrogen receptor (ER) status had relevance to the response of mammary malignancy to chemotherapy. Autophagy has emerged as an important cellular mechanism of tumor cells in response to anticancer therapy. The aim of this study is to investigate whether gemcitabine induces autophagy, and more importantly, whether such autophagy is functional relevant to the therapeutic effects of gemcitabine in breast cancer cells in relation to the ER status. In our study, autophagy was induced both in ER^+^ MCF-7 and ER^−^ MDA-MB-231 cells by gemcitabine markedly, while the autophagy plays distinct roles – cytoprotective in ER^−^ MDA-MB-231 and cytotoxic in ER+ MCF-7 cells. Gemcitabine treatment leads to the activation of ERα-ERK-P62 signal pathway in MCF-7 cells which may augment the autophagic degradation, thus results in the excessive activation of autophagy and irreversible autophagic cell death eventually. Inhibition of ERα-ERK-P62 cascades in MCF-7 cells by small interfering RNA or PD98059 impairs the autophagic degradation, and leads to “autophagic switch” – from cytotoxic autophagy to cytoprotection. Moreover, stable overexpression of ERα in the ER^−^ BCap37 breast cancer cell line enhances the gemcitabine-induced autophagy flux and switches the autophagic cytoprotection in ER^−^ BCap37 to cytotoxicity effect in ER^+^ BCap37 cells. Our study firstly demonstrated that ER status influences gemcitabine efficacy via modulating the autophagy in breast cancer cells.

## INTRODUCTION

Breast cancer is the most prevalent type of malignancy among American women, which is expected to account for 29% of all new diagnosed cancer cases and the second leading cause of cancer-related death in 2014 [1]. Chemotherapy is one of the principal modalities of clinical breast cancer treatment. As adeoxycytidine analogue [2], gemcitabine has been first recommended in the therapeutic regimens for those breast cancer patients who have failed with the treatment of anthracyclines and taxanes [3]. Moreover, many clinical trials have tested the feasibility of gemcitabine in combination with taxanes and/or anthracyclines as a first-line therapy for advanced and/or metastatic breast cancer [4–8]. Unfortunately, a large proportion of breast cancer patients are unresponsive or finally acquire resistance to gemcitabine, but the mechanisms underlying the chemoresistance are complex and still not entirely clear. It has important clinical significance to shed new light into the cellular response after gemcitabine treatment and potential mechanisms of drug resistance, and to promote the optimal use of gemcitabine in breast cancer patients.

It has been well-established that the status of estrogen receptor (ER) plays a crucial role in tailoring therapeutic strategy. ERα-targeted endocrine treatment is routinely used in ER positive breast cancer patients. Moreover, some studies have revealed that ER status may have relevance to the efficacy of chemotherapy. Retrospective analysis of the Southwest Oncology Group protocol 8814 indicated that disease-free survival improved significantly by chemotherapy for postmenopausal woman with breast cancer, but the magnitude of the extra benefit from adding chemotherapy to endocrine treatment was substantially attenuated when the level of ER expression is high [9]. Three CALGB&US Breast Intergroup trials performed another evaluation of the response to chemotherapy influenced by ER status and showed a significant benefit in the subgroups classified as ER-negative rather than ER-positive patients [10]. But until now, the exact relationship between ER status and chemotherapeutic efficacy is undefined.

Autophagy is an evolutionarily conserved process in eukaryotic cells whereby cytoplasmic components are segregated in autophagosomes and degraded by lysosomes for metabolic intermediates and energy recycling [11]. Currently, growing evidence has linked autophagy to cellular response resulted from anticancer therapies and the autophagy is involved in regulating the treatment efficacy. Both pro-survival [12, 13] and pro-death [14, 15] functions have been attributed to autophagy in anticancer therapy.

In this study, we detected the cytotoxic activity of gemcitabine-induced autophagy in breast cancer cell lines with different ER status (ER positive MCF-7, ER negative MDA-MB-231 and ER^−^ Bcap 37/ER^+^ Bcap 37), and investigated the effect of gemcitabine-induced autophagy after inhibition of ERα-mediated signal pathway in MCF-7 cells. Our study aims to clarify whether ER status is functional relevant to the autophagy induced in breast cancer cells that may be related to the therapeutic efficacy of gemcitabine, and also the potential mechanisms involved.

## RESULTS

### Gemcitabine induces autophagy both in ER positive MCF-7 and ER negative MDA-MB-231 cells

**Figure 1:**
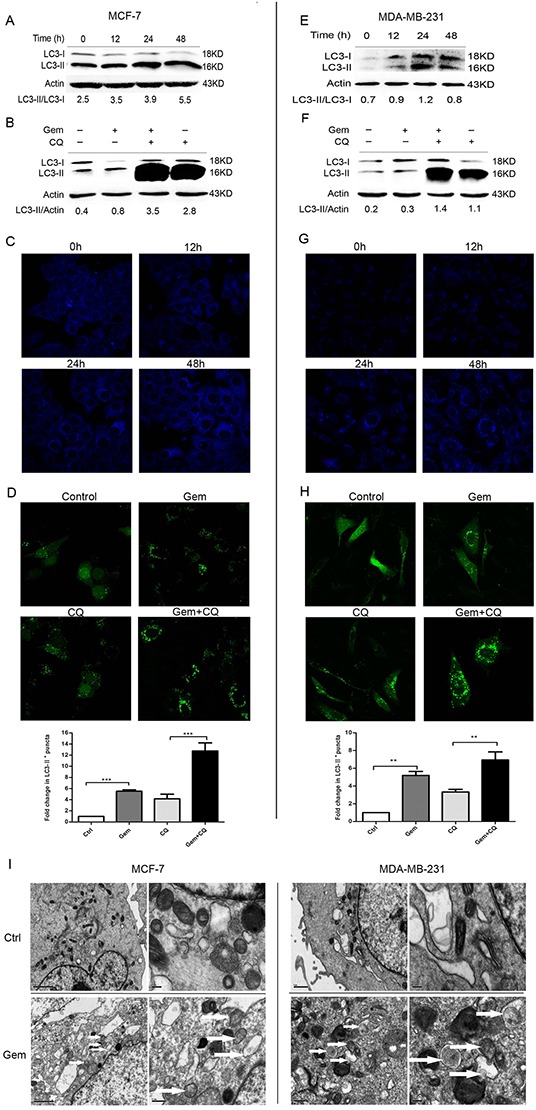
Gemcitabine induced autophagy both in ER-positive MCF-7 and ER-negative MDA-MB-231 cells **A, E.** The levels of LC3-I and LC3-II detected by Western blotting were quantified by densitometry using Image J software. The ratio of LC3-II to LC3-I was evaluated after treated with gemcitabine (MCF-7: 4 μg/ml; MDA-MB-231: 1 μg/ml) for 0 h (control), 12, 24 and 48 h. **B, F.** The ratio of LC3-II to actin was assayed after treated with gemcitabine, chloroquine (CQ) (MCF-7: 2.5 μM; MDA-MB-231: 5 μM), and gemcitabine plus CQ (added before gemcitabine treatment for 1 h) for indicated time (MCF-7: 48 h; MDA-MB-231: 24 h). **C, G.** Both the MCF-7 and MDA-MB-231 cells were exposed to gemcitabine for 0 h (control), 12, 24, 48 h, and treated with MDC (50 μmol/l) for 15 minutes, then fixed. The samples were analyzed by confocal microscope. **D, H.** Cells transiently transfected with GFP-LC3 plasmids were exposed to gemcitabine, CQ, gemcitabine plus CQ for 0 h (control) and 24 h. The confocal microscope was used to analyze GFP fluorescence. The number of LC3-II^+^ puncta was counted in at least 75 cells from random fields and the fold change (mean ± SE) was calculated by normalizing to the amount of control group. *, P<0.05;**, P<0.01;***, P<0.001. **I.** Observation of gemcitabine-induced autophagical vacuoles by transmission electron microscopy (TEM). MCF-7 and MDA-MB-231 cells were treated with gemcitabine for 0 h (control) and 24 h. Autophagical vacuoles with typical double-layer membrane containing remnants of organelles were highlighted by white arrows.

To investigate whether autophagy was activated when cells were exposed to gemcitabine, the lipidation of autophagy marker-microtubule associated protein 1 light chain 3 (MAP1-LC3 or LC3) was examined by western blot to reflect autophagic activity in a certain stable state. The unprocessed form—proLC3 is initially synthesized, and converted into LC3-I, a proteolytically processed form lacking amino acids from the C terminus. When autophagy occurs, LC3-I modified into the phosphatidylethanolamine (PE) -conjugated form, LC3-II, which is the only reliable marker that is specifically associated with autophagosomes. When MCF-7 and MDA-MB-231 cells were exposed to gemcitabine, the ratio of LC3-II/LC3-I was increased in a time dependent manner (Figure 1A, 1E). Due to the degradation of LC3-II in the lysosomes, the increasing of LC3-II/LC3-I ratio is insufficient to justify induction of autophagy by gemcitabine. Thus the relative expression level of LC3-II compared with β-actin was monitored when cells were treated with gemcitabine with or without chloroquine (CQ, autophagic degradation inhibitor) (MCF-7: 2.5 μmol, MDA-MB-231: 5 μmol, added before gemcitabine treatment for 1 h). We identified the concentration of CQ that has little or no toxicity on cells as indicated by MTT assay (data not shown). Compared with the untreated group, LC3-I was converted into LC3-II when cells were treated with gemcitabine; moreover, the relative level of LC3-II in cells treated with gemcitabine plus CQ was significantly higher than that in CQ alone group (Figure 1B, 1F). To further confirm the induction of autophagy by gemcitabine, autophagosomes were visualized using MDC to show the autophagic vacuoles. There was scarcely any bright blue puncta in untreated cells, and the counts of puncta increased significantly with the time of gemcitabine treatment (Figure 1C, 1G). Transient transfection of enhanced green fluorescent protein (EGFP) -LC3 plasmids was performed in order to show the formation of autophagosomes in MCF-7 and MDA-MB-231 cells. The counts of LC3-II^+^ puncta per cell in gemcitabine plus CQ group was statistically higher than that with CQ alone (MCF-7: P<0.001; MDA-MB-231: P<0.01) (Figure 1D, 1H). Ultrastructural analysis showed that numerous autophagic vacuoles of different stages presented in the gemcitabine-treated cells rather than the cells of untreated group (Figure 1I). Taken together, gemcitabine induced autophagy both in MCF-7 and MDA-MB-231 cells, and the activation of autophagy was time dependent.

### Gemcitabine-induced autophagy plays distinct role - cytoprotective in ER^−^ MDA-MB-231 while cytotoxic in ER^+^ MCF-7 cells

**Figure 2:**
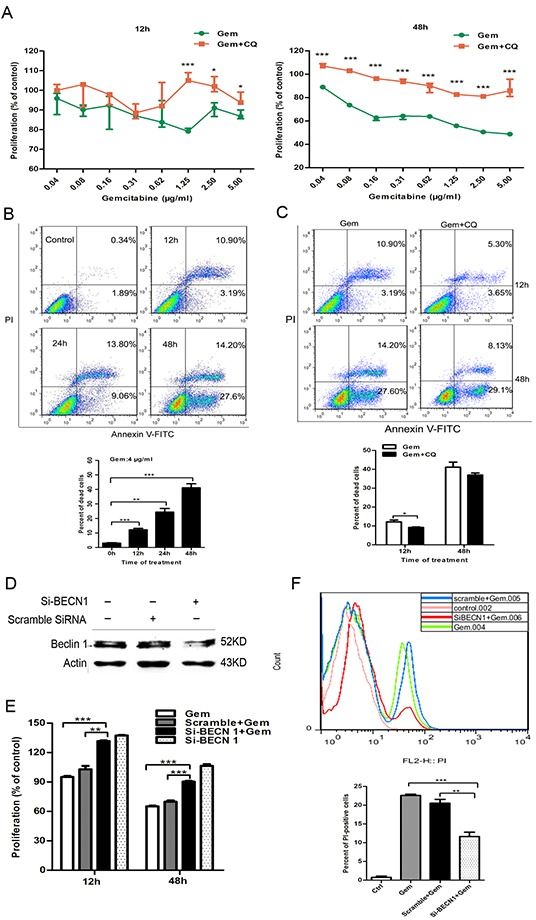
Inhibition of autophagy by CQ and si-BECN1 attenuated the cytotoxicity of gemcitabine in ER-positive MCF-7 cells **A.** The proliferation of MCF-7 cells exposed to gemcitabine alone (0.04-5 μg/ml) or gemcitabine plus CQ (2.5 μM, added before gemcitabine treatment for 1 h) for 12 h and 48 h was detected by MTT assay. The results were expressed as the relative percentage of each group compared with the control. The results (mean ± SE) were from three independent experiments. **B, C.** Cell viability of MCF-7 cells was measured with AV-FITC/PI through flow cytometry after treated with gemcitabine (4 μg/ml) for 0h (control), 12, 24, 48 h or after gemcitabine treatment (4 μg/ml) and gemcitabine plus CQ for 12 h and 48 h. Scatter plots of Annexin V-FITC and PI uptake were shown. The results of statistical analysis were showed in the bar graphs. **D.** The levels of Beclin 1 protein were detected by western blotting after treatment with scramble siRNA or si-BECN1 for 0 h (control) and 48 h. **E.** cells were treated with scramble siRNA or si-BECN1 for 48 h, then incubated with or without gemcitabine (4 μg/ml) for 12 h and 48 h. The proliferation was measured by MTT assay and results were expressed as the relative percentage of each group compared with the control. The results (mean ± SE) were from three independent experiments. **F.** Cells were treated with scramble siRNA or si-BECN1 for 48 h, followed by treatment of 4 μg/ml gemcitabine for 48 h. FACS staining by PI was employed to discriminate between viable and dead cells. In the histograms, PI fluorescent intensity on the X-axis was plotted against the cell number on the Y-axis. The bar graphs showed the percentage of PI-positive cells in the control and post-treated cell populations. The results (mean ± SE) were from three independent experiments. *, P<0.05; **, P<0.01;***, P<0.001.

To clarify the effect of the autophagy induced by gemcitabine on chemotherapy efficacy, drug cytotoxicity on cancer cells before and after pharmacological and genetic inhibition of autophagy was compared. In ER^+^ MCF-7 cells, the proliferation of cells treated with gemcitabine plus CQ for 12 h or 48 h was significantly higher than that with gemcitabine alone (12 h: 0.04-0.62 μg/ml, P>0.05; 1.25 μg/ml, P<0.001; 2.5-5.0 μg/ml, P<0.05; 48 h: 0.04-5.00 μg/ml, P<0.001) (Figure 2A). The viability of MCF-7 was assayed by flow cytometry with Annexin V-FITC (AV-FITC)/PI staining. The results showed that the percentage of dead cells increased after gemcitabine treatment (Figure 2B). When compared to the group with gemcitabine alone, percentage of dead cells in gemcitabine plus CQ group significantly decreased (12 h: P<0.05; 48 h: P>0.05) (Figure 2C) and the double-positive cells was the main population that underwent dramatic decrease. Beclin1 gene silencing was conducted to further investigate the function of autophagy induced by gemcitabine in MCF-7 cells. After treated with siRNA-Beclin1(si-BECN1) for 48 h, Beclin 1 protein level significantly decreased compared to the scramble siRNA group (Figure 2D). The growth of MCF-7 cells treated with si-Beclin1 plus gemcitabine significantly increased when compared to the cells treated with gemcitabine alone or gemcitabine plus scramble siRNA (12 h: P<0.01; 48 h: P<0.001) (Figure 2E). Interestingly, transfection of si-BECN1 alone for 48 h also enhanced the proliferation of gemcitabine-untreated MCF-7 cells, which revealed that Beclin 1 plays a negative role in the survival and proliferation of ER-positive cells. Consistent with the results from CQ, inhibition of the autophagy by si-BECN1 decreased AV-FITC/PI double-positive cells. The percentage of PI^+^ cells in cells treated with si-BECN1 plus gemcitabine was significantly lower than that with gemcitabine alone (P<0.001) or gemcitabine plus scramble siRNA (P<0.01) (Figure 2F). Taken together, these results indicated that the autophagy induction inhibited cell growth and promoted cell death in ER^+^ breast cancer cells when exposed to gemcitabine.

**Figure 3:**
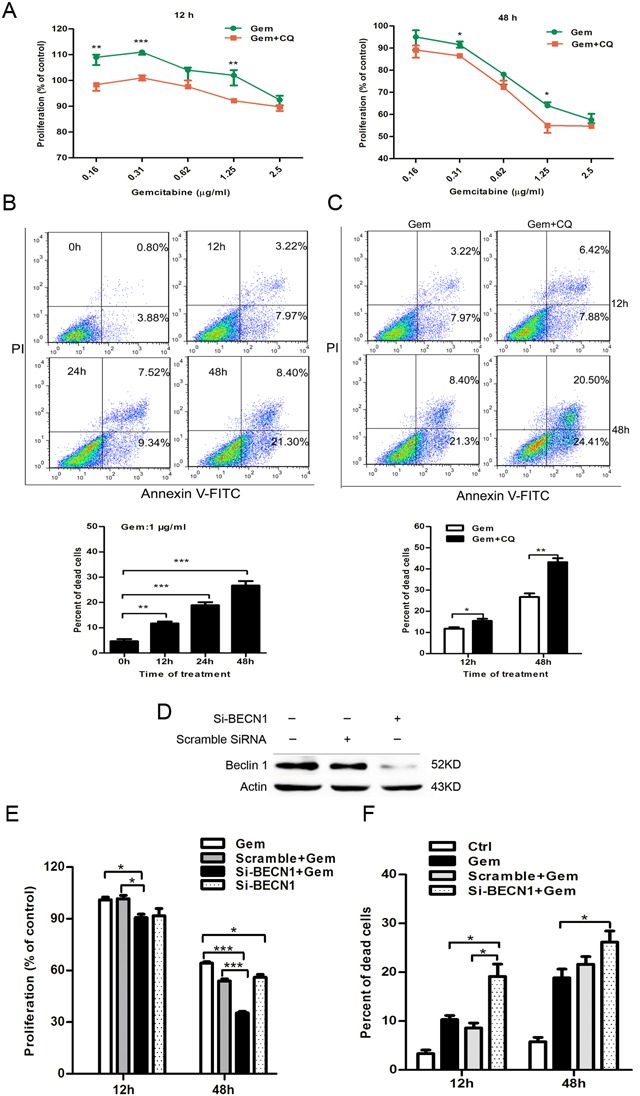
Inhibition of autophagy by CQ and si-BECN1 enhanced the cytotoxicity of gemcitabine in ER-negative MDA-MB-231 cells **A.** The proliferation of MDA-MB-231 cells exposed to gemcitabine alone (0.16-2.5 μg/ml) or gemcitabine + CQ (5 μM, added before gemcitabine treatment for 1 h) for 12 h and 48 h was detected by MTT assay. The results were expressed as the relative percentage of each group compared with the control. The results (mean ± SE) were from three independent experiments. **B, C.** Cell viability of MDA-MB-231 cells was measured with AV-FITC/PI through FACS after treated with gemcitabine (1 μg/ml) for 0, 12, 24, 48 h or gemcitabine plus CQ for 12 h and 48 h. Scatter plots of Annexin V-FITC and PI staining were shown. The results of statistical analysis were showed in the bar graphs. **D.** The levels of Beclin 1 protein were detected by western blotting after treatment with scramble siRNA or si-BECN1 for 0 and 48 h in MDA-MB-231 cells. **E.** Cells were treated with scramble siRNA or si-BECN1 for 48 h, incubated with or without gemcitabine (1 μg/ml) for 12 h and 48 h. The proliferation was measured by MTT assay and the results were expressed as the relative percentage of each group compared with the control. The results (mean ± SE) were from three independent experiments. **F.** MDA-MB-231 cells were treated with or without scramble siRNA or si-BECN1 for 48 h, followed by treatment of 1 μg/ml gemcitabine for 12 h or 48 h, and the cell viability was determined by AV-FITC/PI staining by FACS. The results (mean ± SE) were from three independent experiments. *, P<0.05; **, P<0.01;***, P<0.001.

In contrast to that of MCF-7 cells, inhibition of autophagy increased the cytotoxic outcome of gemcitabine in ER negative MDA-MB-231 cells. Combination of autophagy inhibitor CQ significantly augmented the growth inhibition effect of gemcitabine at 12h and 48h (12 h: 0.16 and 1.25 μg/ml, P<0.01; 0.31 μg/ml, P<0.001; 0.62 and 2.5 μg/ml, P>0.05; 48 h: 0.31 and 1.25 μg/ml, P<0.05; 0.16, 0.62, 2.5 μg/ml, P>0.05) (Figure 3A). The results showed that the percentage of dead cells increased with the time of treatment (Figure 3B). In the group of gemcitabine plus CQ, the percentage of dead cells significantly increased compared to that with gemcitabine alone both at 12 h and 48 h (12 h: P<0.05; 48 h: P<0.01) (Figure 3C). Si-BECN1 resulted in a significant reduction of Beclin 1 protein level in MDA-MB-231 cells (Figure 3D). Compared with the group treated by gemcitabine alone or gemcitabine plus scrambled siRNA, gemcitabine plus si-BECN1 resulted in a more dramatic reduction in cell proliferation (12 h: P<0.05; 48 h: P<0.001) (Figure 3E). In the absence of gemcitabine, si-BECN1 alone for 48 h also inhibited the proliferation of MDA-MB-231 cells, and the inhibition effect of si-BECN1 alone was even stronger than the treatment of gemcitabine alone at the timepoint of 48 h (12 h: P>0.05; 48 h: P<0.05) (Figure 3E), which indicating that Beclin 1 may play a positive role in the survival and proliferation of ER negative MDA-MB-231 cells. Moreover, combination of gemcitabine with si-BECN1 resulted in a more significant increase in the percentage of dead cells when compared to that in group treated with gemcitabine alone (12 h: P<0.05; 48 h: P<0.05) or gemcitabine plus scramble siRNA (12 h: P<0.05; 48 h: P>0.05) (Figure 3F). Thus, the autophagy induced by gemcitabine contributed to the cell growth and played a pro-survival role in ER negative MDA-MB-231, which was distinct to the effect in ER-positive MCF-7.

### Silence of ERα in MCF-7 cells switched gemcitabine-induced autophagy from cytotoxic cytoprotective

**Figure 4:**
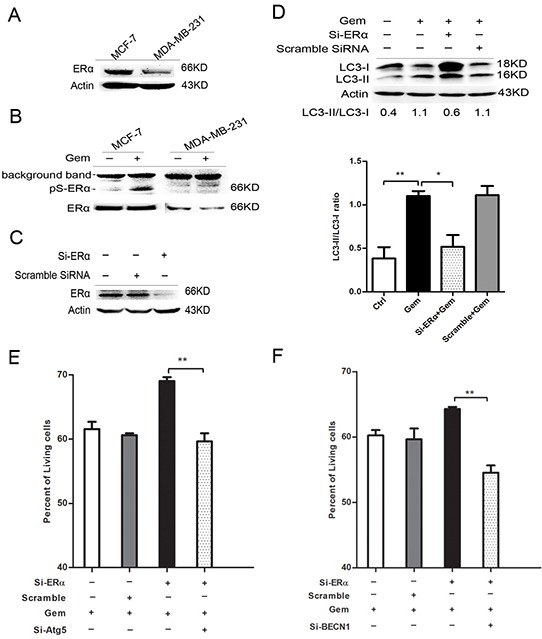
Silence of ERα diminished the gemcitabine-induced autophagy and switched the autophagy from cytotoxic mode to that cytoprotective **A.** ERα expression levels were detected by western blotting in MCF-7 and MDA-MB-231 cells. **B.** The protein levels of ERα and phosphorylation of ERα atser167 were detected after treated with or without gemcitabine both in MCF-7 and MDA-MB-231 cells. **C.** The MCF-7 cells were treated with scramble siRNA or si-ERα for 48 h, then ERα protein level was detected by western blotting. **D.** MCF-7 cells were treated with or without scramble siRNA or si-ERα for 48 h, followed by treatment with 4 μg/ml gemcitabine for another 48 h. The levels of LC3-I and LC3-II detected by western blotting were quantified by densitometry using Image J softwar. The ratio of LC3-II to LC3-I was evaluated in three independent experiments. **E.** MCF-7 cells were treated with scramble siRNA, si-Atg5 and/or si-ERα for 48 h, followed by gemcitabine (4 μg/ml) for 48 h. The percentage of living cells relative to the control group was determined by MTT assay. The results (mean ± SE) were from three independent experiments. **F.** MCF-7 cells were treated with scramble siRNA, si-BECN1 and/or si-ERα for 48 h, followed by gemcitabine treatment (4 μg/ml) for another 48 h. The percentage of living cells relative to the control group was determined by MTT assay. The results (mean ± SE) were from three independent experiments. *, P<0.05; **, P<0.01;***, P<0.001.

The result of immunoblotting confirmed that ERα level of MCF-7 cells is higher than that of MDA-MB-231 cells (Figure 4A). Phosphorylation of ERα atser167 was induced remarkably when ER^+^ MCF-7 cells were exposed to gemcitabine (Figure 4B). Transient transfection of ERα siRNA in MCF-7 cells caused >80% knockdown of ERα (Figure 4C). ERα knockdown for 48 h before gemcitabine exposure resulted in an obvious increase both in LC3-II and LC3-I when compared to that with gemcitabine alone or scramble siRNA plus gemcitabine, while the ratio of LC3-II/I was significantly decreased (P<0.05) (Figure 4D), indicating that the attenuation of autophagic flux. Then we explored the role of autophagy in ERα-silenced MCF-7 cells that treated by gemcitabine. Inhibition of the autophagy by si-Atg5 augmented the inhibitory effect of gemcitabine on cell growth of MCF-7 transfected with si-ERα (P<0.01) (Figure 4E), indicating autophagy induced by gemcitabine played a cytoprotective role in ERα-silenced MCF-7 cells, which confirmed the existence of “autophagic switch”. Similar result was observed using si-BECN1 to inhibit the autophagy in gemcitabine-treated ERα-silenced MCF-7 cells (P<0.01) (Figure 4F).

### Overexpression of ERα enhanced the gemcitabine-induced autophagy and switched the cytoprotection in BCap37 breast cancer cells

**Figure 5:**
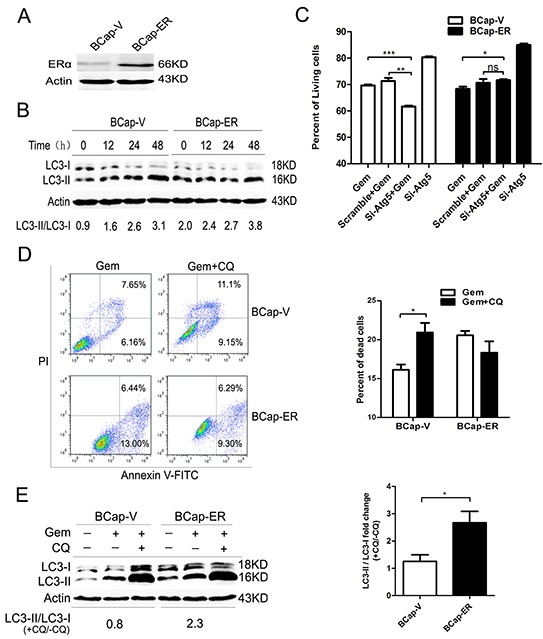
Overexpression of ERα enhanced the gemcitabine-induced autophagy and switched the autophagy from cytoprotective mode to that cytotoxic in the ER-negative BCap-37 breast cancer cells **A.** ERα expression levels were detected by Western Blotting in BCap-37 breast cancer cells transfected with empty vector (BCap-V) or pIRES-ERa expression vector (BCap-ER). **B.** BCap-V and BCap-ER cells were treated with gemcitabine (75 μg/ml) for 0h (control),12, 24 and 48h. The levels of LC3-I and LC3-II detected by Western blotting were quantified by densitometry using ImageJsoftwar, and the ratio of LC3-II to LC3-I was evaluated. **C.** BCap-V and BCap-ER cells were treated with scramble siRNA or si-Atg5 for 48 h, then incubated with or without gemcitabine (75 μg/ml) for 48 h. The percentage of living cells relative to the control group was determined by MTT assay. The results (mean ± SE) were from three independent experiments. **D.** Cell viability of BCap-V and BCap-ER cells was measured with AV-FITC/PI through a flow cytometer after treated with gemcitabine (75 μg/ml) or gemcitabine plus CQ (5 μM, pretreatment for 1 h) for 48 h. Scatter plots of Annexin V-FITC and PI uptake were shown. The results of statistical analysis were showed in the bar graphs. **E.** The ratio of LC3-II to LC3-I was assayed after treated with gemcitabine, and gemcitabine plus CQ for 48 h. Then the fold change of LC3-II/LC3-I ratio in the gemcitabine plus CQ group compared with that gemcitabine alone was evaluated in BCap-V and BCap-ER cells respectively. The results (mean ± SE) were from three independent experiments. *, P<0.05;**, P<0.01;***, P<0.001.

To eliminate the possible deviation due to the genetic alterations between different breast cancer cell lines, a pair of isogenic ERα^+^ BCap-ER /ERα^−^ BCap-V (control) breast cancer cell lines was used. It was built through stable transfection of ERα expression vector or empty vector into ERα^−^ BCap37 breast cancer cells. The immunobloting confirmed that ERα level in BCap-ER cells was dramatically higher than that of BCap-V cells (Figure 5A). When BCap-V and BCap-ER cells were exposed to gemcitabine, the ratio of LC3-II/LC3-I was increased in a time dependent manner (Figure 5B), indicating the autophagy activation. Inhibition of the autophagy by si-Atg5 augmented the inhibitory effect of gemcitabine on the BCap-V cell growth (P<0.001), but the inhibitory effect was decreased in BCap-ER cells (P<0.05) (Figure 5C), indicating the distinct role of gemcitabine-induced autophagy in BCap-ER cells and parental BCap-V cells. FACS analysis with Annexin V-FITC/PI staining was used to detect the viability of BCap-V and BCap-ER cells treated with gemcitabine before and after inhibition of autophagy by CQ. The results showed that the percentage of dead cells significantly increased in BCap-V cells pretreated with CQ before gemcitabine exposure (Figure 5D, P<0.05), while decreased in BCap-ER cells (Figure 5D, P>0.05). To reflect the level of autophagic flux and the relative autophagic degradation, the fold change of LC3-II/LC3-I ratio between gemcitabine plus CQ group with gemcitabine alone group was shown. Expectedly, the LC3-II/LC3-I fold change was more significant in BCap-ER cells than that in ERα negative BCap-V cells. Collectively, overexpression of ERa enhanced the gemcitabine-induced autophagy and switched the autophagic cytoprotection in ER negative BCap37 cells to cytotoxicity in BCap-ER, which provided further evidence confirming that ER status could influence gemcitabine efficacy in breast cancer cells via modulating the autophagy process.

### Inhibition of ERα/ERK/P62 cascades induced “autophagic switch” in ER positive MCF-7 cells that exposed to gemcitabine

**Figure 6:**
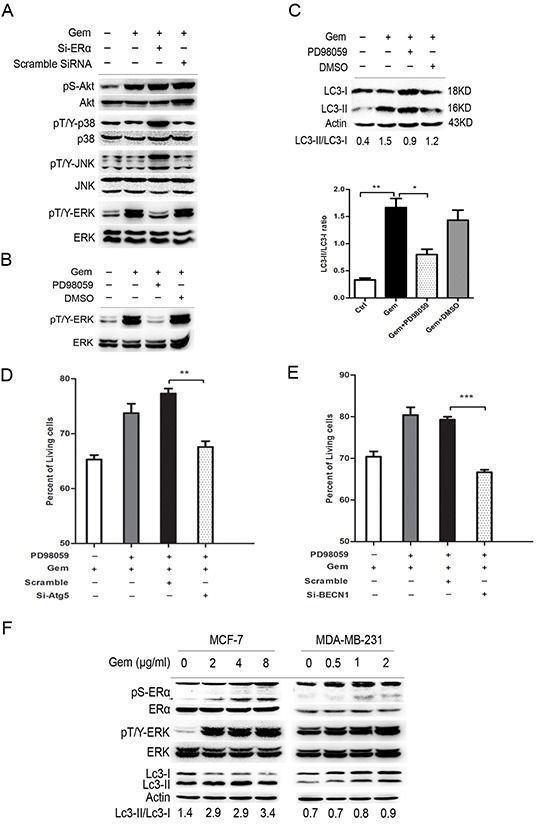
ERK was a downstream regulator of ERα-mediated autophagic cell death and inhibition of ERK also induced the “autophagic switch” in MCF-7 cells **A.** MCF-7 cells were treated with or without scramble siRNA or si-ERα, followed by gemcitabine treatment (4 μg/ml) for 48 h. Then total cell lysates were subjected to immunoblot analysis with indicated antibodies. The data represented a typical result and the experiments were conducted three times with similar results. **B.** The phosphorylated levels of ERK at Thr202/Tyr204 were detected by immunoblot after treatment with gemcitabine combined with PD98059 or DMSO (added before gemcitabine treatment for 1 h). **C.** MCF-7 cells were treated by gemcitabine (4 μg/ml) combined with PD98059 or DMSO for 48 h. The levels of LC3-I and LC3-II detected by Western blotting were quantified by densitometry using ImageJ software. The ratio of LC3-II to LC3-I was evaluated in three independent experiments. **D.** MCF-7 cells were treated with scramble siRNA or si-Atg5 for 48 h, followed by gemcitabine and PD98059 for another48 h. The percentage of living cells relative to the control group was detected by MTT assay. **E.** MCF-7 cells were treated with scramble siRNA or si-BECN1 for 48 h, followed bygemcitabine and PD98059 for another48 h. The percentage of living cells relative to the control group was detected by MTT assay. **F.** MCF-7 and MDA-MB-231 cells were treated by gemcitabine with different concentrations (MCF-7: 0, 2, 4, 8 μg/ml; MDA-MB-231: 0, 0.5, 1, 2 μg/ml), and the phosphorylation of ERα (ser167), ERK (Thr202/Tyr204) and expression of LC3-I and LC3-II were detected. All the results (mean ± SE) were from three independent experiments.**, P<0.01;***, P<0.001.

To further explore the signal pathway that mediated the “autophagic switch”, we detected the expression level of downstream molecules, including MAPK family and AKT. As showed in Figure 6A, gemcitabine obviously upregulated the phosphorylation of extracellular signal-regulated kinase 1/2 (ERK1/2), but not JNK, P38 and AKT, implying that ERK1/2 phosphorylation might be involved in the ERα-mediated autophagic cell death induced by gemcitabine in MCF-7 cells, while si-ERα significantly downregulated gemcitabine-induced ERK phosphorylation. An ERK1/2 inhibitor, PD98059, was used to verify the role of ERK1/2 in the gemcitabine-induced autophagy in ER positive MCF-7 cells. The phosphorylated ERK1/2 activated by gemcitabine was dramatically inhibited by PD98059 (Figure 6B), a marked accumulation of both LC3-I and LC3-II was induced when PD98059 was added to gemcitabine-treated MCF-7 cells, while the LC3-II/LC3-I ratio was significantly decreased (P<0.05) (Figure 6C). Moreover, similar to that in ERα-silenced MCF-7 cells, inhibition of the autophagy by si-BECN1 or si-Atg5 enhanced the inhibitory effect of gemcitabine on cell growth of MCF-7 cells treated by PD98059, indicating that gemcitabine-induced autophagy played a cytoprotective role in ERK1/2-inhibited MCF-7 cells (Left: P<0.01; Right: P<0.001) (Figure 6D, 6E). To further prove the activation of ERα-ERK1/2 cascade and its role in the gemcitabine-induced autophagy in ER^+^ MCF-7 cells, we analyzed phosphorylated ERα (P-ERα), P-ERK1/2, and the ratio of LC3-II/I with the increase of gemcitabine concentration. The phosphorylation of ERα at ser167 was activated in a concentration-dependent manner (Figure 6F). Furthermore, both in MCF-7 and MDA-MB-231 cells, the trend of ERK1/2 phosphorylation was correlated with the LC3-II/LC3-I ratio (Figure 6F). All these results suggested that ERK1/2 might function as a downstream regulator in ERα-mediated autophagic cell death that induced by gemcitabine.

**Figure 7:**
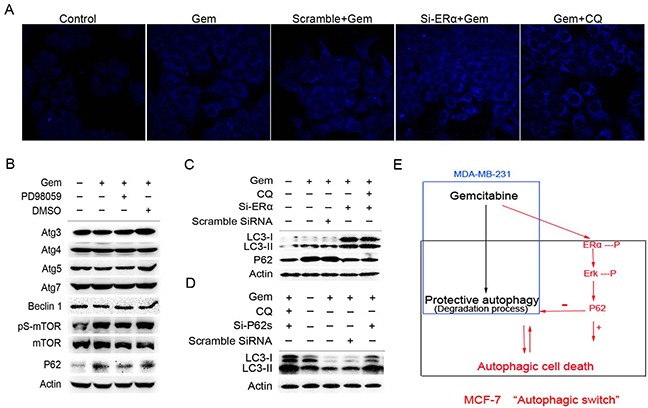
Excessive activation of the P62-mediated autophagic degradation by the phosphorylated ERα-ERK cascades may lead to the autophagic cell death induced by gemcitabine in ER positive MCF-7 cells **A.** MCF-7 cells that exposed to scramble siRNA or si-ERα for 48 h were treated by gemcitabine alone (4 μg/ml) or gemcitabine + CQ (2.5 μmol/L, added before gemcitabine treatment for 1 h) for another 48 h. Then the cells were treated with MDC (50 μmol/L) for 15 minutes and fixed. The samples were analyzed by confocal microscope. **B.** MCF-7 cells were treated by gemcitabine, gemcitabine+ PD98059 (30 μmol/L, added before gemcitabine treatment for 1 h) or DMSO for 48 h. Then total cell lysates were subjected to immunoblot analysis with indicated antibodies. The data represented a typical experiment conducted three times with similar results. **C.** MCF-7 cells were exposed to scramble siRNA or si-ERα for 48 h, and treated by gemcitabine or gemcitabine + CQ for another 48 h. The expression levels of LC3-I/II and P62 proteins were evaluated by Western blotting. β-actin was used as a loading control. **D.** MCF-7 cells were exposed to scramble siRNA or si-P62 for 48 h, and treated by gemcitabine or gemcitabine + CQ for another 48 h. The expression of LC3-I and LC3-II proteins was detected by immunoblot analysis and actin was used as a loading control. **E.** Working model for the “autophagic switch”. In ER negative MDA-MB-231, gemcitabine induced the protective autophagy. While in ER positive MCF-7 cells, the phosphorylation of ERα-ERK cascades induced by gemcitabine activated the P62-mediated autophagic degradation excessively, and as a result, it may lead to the collapse of cellular function and autophagic cell death. Inhibition of ERα-ERK cascades removed the excessive activation from the autophagic degradation process induced by gemcitabine, thus resulted in the autophagy that the degree was under a certain threshold, and was beneficial for the adaptation of cells in unfavorable conditions, thereby contributing to the cells survival.

We then asked what molecules are involved in the autophagic process affected by the activation of ERα/ERK1/2 cascades in ER^+^ MCF-7 cells exposed to gemcitabine. Both of si-ERα and CQ markedly increased the counts of bright blue puncta in the gemcitabine treated MCF-7 cells (Figure 7A). Inhibition of ERK1/2 by PD98059 had no effect on the expression of autophagy related genes except for P62, which usually participates in the autophagy degradation. Interestingly, P62 was markedly increased when autophagy was activated by gemcitabine, while it decreased significantly when cells were treated with gemcitabine plus PD98059 (Figure 7B). Thus we hypothesized that P62 might act as the downstream of ERα-ERK1/2 cascades and promote autophagic degradation process. In two groups of ERα-silenced MCF-7 cells that treated by gemcitabine with or without CQ, both of LC3-I/II proteins were markedly accumulated (Figure 7C), implying that knockdown of ERα played a similar role with CQ. It was noteworthy that gemcitabine induced the expression of P62, while P62 was decreased markedly in the cells transfected of si-ERα. Si-ERα combined with CQ to inhibit autophagic degradation did not render P62 increase when compared to MCF-7 cells with si-ERα alone after gemcitabine treatment (Figure 7C). Expectedly, knockdown of P62 by targeted siRNAs led to the remarkable accumulation of both LC3-I and LC3-II proteins in the gemcitabine-treated MCF-7 cells (Figure 7D). These results revealed that P62 not only serves as a readout of autophagic degradation, but also acts as a downstream molecule of ERα-ERK signaling pathway in MCF-7 cells treated by gemcitabine, and exerted an indispensable function in the autophagic degradation.

## DISCUSSION

Retrospective analysis of some clinical trials reveals that ER status of breast cancer patients seems to correlate with chemotherapeutic efficacy [16–18], but few studies have focused on the exact relationship between them. Our results in the present study confirmed that the autophagy induced exerts distinct effects on gemcitabine-treated cells between ER positive MCF-7 cells and ER negative MDA-MB-231 cells. The significance of autophagy on drug efficacy is investigated by comparing the growth inhibitory effect of gemcitabine on breast cancer cells before and after inhibition of autophagy using chloroquine and si-Beclin1. Chloroquine is a safe drug which has been used extensively for malaria treatment and clinical safety data are available. Beclin 1/Atg 6 is an essential component in the autophagy progress which signals the onset of autophagy [19]. By binding to the anti-apoptotic protein BCL2, Beclin 1-dependent autophagy is inhibited [20], autophagy is induced by the release of Beclin 1 from BCL2 by pro-apoptotic BH3 proteins [21, 22]. In ER^−^ MDA-MB-231, Beclin 1-mediated autophagy attenuates the cytotoxicity of gemcitabine. Interestingly, treatment with si-Beclin1 alone also obviously inhibits the proliferation of MDA-MB-231 cells. In contrast, Beclin1-mediated autophagy accelerates cell death and improves the cytotoxic outcome of gemcitabine to MCF-7 cells. Overall, Beclin 1-mediated autophagy was pro-survival in ER-negative MDA-MB-231 cells, while pro-death in ER-positive MCF-7 cells when exposure to gemcitabine. Beclin 1 gene plays counter roles in the proliferation of ER^+^ and ER^−^ cells rather than a single role that acts as a negative regulator of mammary cell growth [23].

One study published by Sunga Choi demonstrates that cordycepin, found in Cordyceps spp., effectively triggers autophagic cell death that was ER-independent in human MCF-7 and MDA-MB-231 cells [24]. In the present study, silence of ERα by siRNA in MCF-7 downregulates the autophagy flux activated by gemcitabine, and switches the function of the autophagy to cytoprotective. It has been reported that phosphorylation at specific amino acid residues can alter ER activity [25–28]. Ser167, which maps to serine residue in the activation function 1 (AF-1) domain, is a crucial phosphorylation site of ERα and plays an important role in the modulation of ER activity. Multiple studies demonstrated that phosphorylation of ERα at ser167 is closely related with curative effect, indicative of longer disease-free and overall survival in breast cancer patients [29–31]. In our study, phosphorylation of ERα at ser167 is obviously augmented in MCF-7 cells after gemcitabine treatment, revealing that gemcitabine is able to stimulate ERα activation in ligand-independent manner. In addition to the transcription action mediated by the canonical genomic ER, many studies demonstrated the existence of rapid ERα-mediated actions, which occur in cell membrane and mitochondria, revealing that conventional ER can also locate in cell membrane (defined as membrane estrogen receptor, mER) [32, 33]. It is believed that ER proteins shuttle between the cytoplasm and nucleus, and possess two different patterns of action. Distinguishing from the nuclear-initiated actions (genomic pattern) of ER, the activation of extra-nuclear ER mediated the membrane-initiated steroids signaling (non-genomic pattern), which involves rapid activation of manifold downstream signaling molecules, such as mitogen-activated protein kinases (MAPKs), adenylyl cyclase (AC), protein kinase C (PKC) and Akt [34]. In our study, knockdown of ERα attenuates the autophagic flux and switches the function of autophagy to cytoprotective, indicating that ERα plays an important role in the maintaining of autophagic cell death in gemcitabine-treated MCF-7 cells. Inhibition of the ERK by PD98059 induces the change of autophagic protein expression, which was similar to the effect of ERα knockdown in MCF-7, suggesting that activation of ERK1/2 phosphorylation may participate in the ERα-mediated signaling pathway for autophagic cell death.

The P62 protein was generally used as a protein marker that its expression was negatively correlated with autophagy activation [35]. It contains an LC3 connecting motif as well as an ubiquitin binding domain, linking ubiquitinated proteins with the autophagic machinery [19]. The protein complexes incorporate into autophagosomes, and then degrade in autolysosomes for biomacromolecule recycling. In addition to serve as a readout of autophagic degradation, it was worth noting that P62 was upregulated in some situations where the autophagic flux was increased, indicating that it may have additional functions in autophagy process that need to be further studied [36, 37]. Zheng Q et al. studied the role of autophagy and P62 protein in protection of cardiac proteinopathy *in vitro* and in mice model. Their results showed that P62 protein mediates aggresome formation and triggers the activation of selective autophagic degradation [38]. In our study, the level of P62 protein was markedly increased when autophagy flux was activated by gemcitabine in MCF-7 cells, while decreased if ERα-ERK cascades was knocked down or chloroquine inhibited the autophagic degradation process. Moreover, silence of P62 by targeted siRNAs induced the accumulation of both LC3-I and LC3-II proteins. It suggested that P62 protein acts as the downstream regulatory molecule of ERα-ERK1/2 cascades and plays an essential function in autophagic degradation program in ER positive MCF-7 cells treated with gemcitabine.

Autophagy enables cells to maintain homeostasis in unfavorable conditions, thereby contributing to cell survival. However, if the insult is too severe and the activation of the autophagic pathway beyond a certain threshold, it may cause collapse of cellular functions that results in cell death directly [39]. Samaddar et al. studied autophagosomes formation in the surviving MCF-7 cells after antiestrogen treatment, and they hypothesized that whether autophagy promotes survival or cell death may be dependent on the number of autophagosomes in each cell, resulting in a threshold limit [40]. We hypothesized that the activation of ERα/ERK/P62 cascades in gemcitabine-treated MCF-7 cells might excessively augment the P62-mediated autophagic degradation, and as a result, the autophagy exceeds certain threshold where cell death become inevitable. Wilson et al. firstly demonstrated the existence of an “autophagic switch”. They showed that 1, 25 D3 appear to switch the cells from a cytoprotective to a cytotoxic mode of autophagy in radiation treated ZR-75-1 human breast tumor cells [41]. Aside from the superficial functional differences between cytoprotective and cytotoxic autophagy, there were no absolute quantitative, biochemical or molecular parameters that have been identified to distinguish between the two forms of autophagy in response to anticancer therapy [42]. In the present study, we firstly confirmed that ERα affects the activation level and function of gemcitabine-induced autophagy in breast cancer cells, and modulation of ERα expression is able to induce “autophagic switch” from cytotoxic to cytoprotective mode.

Collectively, our study firstly demonstrated that inhibition of ERα/ERK cascades in gemcitabine-treated MCF-7 cells weakens the P62-mediated autophagic degradation, and induces the “autophagic switch”-from the cytotoxic autophagy to cytoprotective autophagy. It suggested that combination of gemcitabine with autophagy promoter (like Vit D) in the patients with high ER expression, or with autophagy inhibitor (like hydroxylchloroquine) in patients with negative/low ER expression will be a feasible strategy that may have clinical significance for breast cancer patients with gemcitabine treatment. However, cell fates in response to chemotherapy were results of multiple mechanisms, including autophagy, apoptosis, cell cycle arrest and so on. And there was close relationship among these mechanisms, further studies are still needed to clarify the molecular mechanisms involved.

## MATERIALS AND METHODS

### Cell lines and reagents

Monolayer culture of MCF-7 cells (from American Type Culture Collection) were maintained in DMEM supplemented with 10% fetal bovine serum and 100 μg/ml streptomycin, 100 units/ml penicillin in a humid incubator with 5% CO_2_ at 37°C. MDA-MB-231 cells (from American Type Culture Collection) were cultured at 37°C in L-15 medium supplemented with 10% FBS, 100 μg/ml streptomycin and 100 units/ml penicillin. BCap37 breast cancer cell line, which first established in China, was kindly provided by Prof. Weimin Fan (Zhejiang University, Hangzhou). BCap37 cells were transfected with pIRES-ERα expression vector (BCap-ER) and cultured in RPMI 1640 supplemented with 10% FBS and 250 μg/ml geneticin (G418, Sigma). BCap37 cells transfected with empty vector (BCap-V) were cultured in RPMI 1,640 medium containing 10% FBS [43].

Gemcitabine (diluted in 0.9% normal saline, G6423), Chloroquinediphosphate salt (diluted in double distilled water, C6628), monodansylcadaverine (MDC, diluted in PBS, 30432) was purchased from Sigma-Aldrich. The Annexin V Apoptosis Detection Kit FITC (88-8005) and Propidiumlodide Staining Solution (00-6990) was from eBioscience, Inc. PD98059 (s1177) was from Selleck.cn. Anti-LC3B Antibody (L7543), Anti-ATG4B Antibody (A2981), Anti-ATG5 Antibody (A0731), Anti-ATG7 (A2856) produced in rabbit was from Sigma-Aldrich. Beclin1/ATG6 Antibody (Rabbit polyclonal, NB500-249), p62/SQSTM1 Antibody (Mouse Monoclonal, NBP2-23490) was from Novus Biologicals. mTOR Antibody(2972s), Phospho-mTOR Antibody(Ser2448), Akt Antibody (4691s), Phospho-Akt (Ser473) Antibody (4060s), p44/42 MAPK (Erk1/2) Antibody (4695), Phospho-p44/42 MAPK (Erk1/2) (Thr202/Tyr204) Antibody (4370s), SAPK/JNK Antibody (9252s), Phospho-SAPK/JNK (Thr183/Tyr185) Antibody (4668), p38 MAPK Antibody (9212), Phospho-p38 MAPK (Thr180/Tyr182) Antibody (9211), Phospho-Estrogen Receptor α(Ser167) Antibody (5587P), Anti-ATG3 Antibody (3415P) produced in rabbit was from Cell Signaling Technology. ERα Antibody (sc-542) – an affinity purified rabbit polyclonal antibody and the secondary antibodies – HRP conjugated goat anti-rabbit and goat anti-mouse IgG was from Santa Cruz Biotechnology, Inc.

### Cell proliferation and viability assay

The effect of gemcitabine on MCF-7 and MDA-MB-231 cell proliferation was analyzed by the MTT colormetric method. Cells seeded into 96-well flat-bottomed plates were allowed to attach overnight at 37°C then treated with Gemcitabine with or without chloroquine with different concentrations for the indicated time. Afterwards, 20 μl MTT (5 mg/ml) was added into each well for 4 h incubation at 37°C, and 150 μl DMSO added into each well dissolve any blue-purple crystals of formazan. The plates were agitated for 15 minutes. Absorbance values at 490 nm were detected with a model 680 MicroPlate Reader (Bio-Rad). Cell growth inhibition was calculated on the basis of the following formula: Proliferation (% of the control) = [A490 (experimental group) – A490 (negative control group)/A490 (control group) – A490 (negative control group)] x 100. For the viability assay, cells were seeded at 2×105 cells per well in 6-well plates, allowed to attach overnight at 37°C, and were treated for the indicated concentration. After the scheduled time, Cells were trypsinized and dyed with Annexin V-FITC and Propidiumlodide (PI) performed according to the instruction of the manufacturer. Then the cells viability was analyzed by flow cytometry (BD FACSCalibur). Tests were repeated three times independently.

### Western blot analysis

Cells were seeded in 6-well plates and treated with the agents for the specified time interval. After treatment, both detached and attached cells were collected by centrifugation, and whole cell lysates were obtained after adding 1× lysis buffer (1×PBS pH 7.6, 1% NP-40, 0.1% sodium dodecyl sulfate and 0.5% sodium deoxycholate supplemented with inhibitor cocktails) to the cell deposit for 30 minutes on ice afterwards. Then the cell lysates were cleared by centrifugation at 12,000×g for 15 minutes at 4°C, and the supernatant fraction was collected for immunobloting analysis. About 30 μg total protein of each group were separated by 10% or 15% SDS-PAGE and transferred onto a PVDF membrane (Bio-Rad). The PVDF membranes were blocked by 5% nonfat dry milk diluted in tris-buffered saline-Tween 20 (TBST) for 90 minutes at room temperature. Afterwards, the membranes incubated in primary antibodies diluted in 5% nonfat dry milk in TBST overnight with light agitation at 4°C, washed with TBST three times, every ten minutes, then incubated with light agitation in secondary antibodies diluted in 5% nonfat dry milk in TBST for 2 h at room temperature. Washing three times with TBST and three times using TBS was carried out before the membrane were analyzed with electrochemiluminescience (ECL, Bio-Rad).

### Visualization of monodansylcadaverine-labeled vacuoles

In order to analysis autophagy induced by gemcitabine in cells, MDC—a fluorescence dye incorporated selectively into autophagosomes was used to visualize autophagosomes. MDC was stored at −20°C with a stock solution of 100 mM that made in DMSO. Cells on cover slips in 24-well plates were treated for the indicated time, then fresh medium containing 50 μmol/L MDC was added into the cells for 15 minutes. A freshly made 4% formaldehyde solution in PBS was used to fix the cells for 10 minutes at room temperature. The cover slips were examined under a BX61W1-FV1000 confocal microscope system (Olympus, Japan).

### GFP-LC3 plasmid transfection

The GFP-LC3 plasmid was a kind gift from Professor Wang Xiaojian (The Institute of Molecular Immunology, Zhejiang University, China). The plasmids were transfected into cells transiently by Lipofectamine 2000TM (Invitrogen) according to the instructions of the manufacturer, and the fluorescence of GFP was assessed with BX61W1-FV1000 confocal microscope (Olympus, Japan). The LC3-II^+^ puncta number was counted manually in confocal images from random fields to quantify the GFP-LC3 tagged autophagosomes.

### Transmission electron microscope (TEM)

Cells were seeded in six-well plates and allowed to attach by overnight incubation at 37°C. The cells were treated with or without gemcitabine of the concentration of the specific for 24 h. The treated cells were fixed in ice-cold 2.5% glutaraldehyde for 1 h, and then washed with 0.1 M PBS, fixed again in 1% osmium tetroxide for 1 h. Next, the samples were washed with ice-cold double distilled water and stained with 4% uranyl acetate for 30 min. Afterwards, the samples were dehydrated through a graded series of ethanol solutions (range from 50% to 100%), and embedded in Epon. Ultrathin sections (120 nm) were stained with 4% uranyl acetate for 20 min and lead citrate for 5 min, and then examined in the TECNAI 10 transmission electron microscope (Philips, Holland) at 60 kV.

### RNA interference

Cells were seeded in 6-well plates and transfected at 50% confluency with either scramble siRNA or targeted siRNA using Interferin SiRNA Transfection Reagent according to the recommendation of manufacturer. After 48 h transfection, cells were treated with or without Gemcitabine for the indicated time. Then the cells were collected and processed for western blot analysis or AnnexinV-FITC/PI apoptosis assay. The si-ERα targeting sequence was GGC CAA AUU CAG AUA AUCG or AAU GAU GAA AGG UGG GAU A; The si-BECN1 targeting sequence was UUC AAC ACU CUU CAG CUC AUC AUCC; The si-Atg5 targeting sequence was CCU UUG GCC UAA GAA GAA ATT dTdT; The si-P62 targeting sequence was CCA GAC UAC GAC UUG UGU Att or GUG UGA AUU UCC UGA AGA Att or CCA UCC UGU UAA AUU UGU Att. Off-target effects by siRNA were verified by using appropriate nontargeting scrambled siRNAs in all of our transfection experiments as negative control. All siRNAs were obtained from GenePharma (China). InterferinSiRNA Transfection Reagent was from PolyPlus Transfection, Inc.

### Statistical analysis

Statistical analysis was performed using Student's *t* test, analysis of variance (anova) using SPSS (v 16.0). P≤0.05 was considered to be statistically significant.

## Abbreviations

CQ: chloroquine
ER: estrogen receptor
FITC: fluorescein isothiocyanate
GFP: green fluorescent protein
MDC: monodansylcadaverine
MTT: 3-(45-dimethylthiazol-2yl)-25-diphenyltetrazolium bromide
MAP1-LC3: microtubule-associated protein 1 light chain 3
PI: Propidiumlodide
siRNA: small interfering RNA
TEM: Transmission electron microscopy
